# Disseminated Talaromyces marneffei infection initially presenting as cutaneous and subcutaneous lesion in an HIV-Negative renal transplant recipient: a case report and literature review

**DOI:** 10.1186/s12879-024-09351-8

**Published:** 2024-05-06

**Authors:** Shanshan Yang, Lixin Lou, Shuhong Ma, Haoliang Wang, Lanxiang Rong, Ying Liu, Kaiyu Zhang, Qing Ai, Xu Shi

**Affiliations:** 1 Department of Laboratory Medicine, Lequn Branch, The First Hospital of Jilin University, No. 3302 Jilin Road, Changchun, 130031 Jilin Province China; 2 Department of Infectious Diseases, The First Hospital of Jilin University, Changchun, 130021 Jilin China; 3 Department of Hand Surgery, The First Hospital of Jilin University, Changchun, 130021 Jilin China

**Keywords:** *Talaromyces marneffei*, Renal transplantation, Gastrointestinal bleeding, Opportunistic fungal infection, Antifungal therapy

## Abstract

**Background:**

The incidence of *Talaromyces marneffei* (*T. marneffei*) infection has increased in recent years with the development of organ transplantation and the widespread use of immunosuppressive agents. However, the lack of clinical suspicion leading to delay or misdiagnosis is an important reason for the high mortality rate in non-human immunodeficiency virus (HIV) and non-endemic population. Herein, we report a case of disseminated *T. marneffei* infection in a non-HIV and non-endemic recipient after renal transplant, who initially presented with skin rashes and subcutaneous nodules and developed gastrointestinal bleeding.

**Case presentation:**

We describe a 54-year-old renal transplantation recipient presented with scattered rashes, subcutaneous nodules and ulcerations on the head, face, abdomen, and right upper limb. The HIV antibody test was negative. The patient had no obvious symptoms such as fever, cough, etc. Histopathological result of the skin lesion sites showed chronic suppurative inflammation with a large number of fungal spores. Subsequent fungal culture suggested *T. marneffei* infection. Amphotericin B deoxycholate was given for antifungal treatment, and there was no deterioration in the parameters of liver and kidney function. Unfortunately, the patient was soon diagnosed with gastrointestinal bleeding, gastrointestinal perforation and acute peritonitis. Then he rapidly developed multiple organ dysfunction syndrome and abandoned treatment.

**Conclusions:**

The risk of fatal gastrointestinal bleeding can be significantly increased in kidney transplant patients with *T. marneffei* infection because of the long-term side effects of post-transplant medications. Strengthening clinical awareness and using mNGS or mass spectrometry technologies to improve the detection rate and early diagnosis of *T. marneffei* are crucial for clinical treatment in non-HIV and non-endemic population.

## Background

*Talaromyces marneffei* (*T. marneffei*), initially named *Penicillium marneffei*, was isolated from the liver of bamboo rats [1]. It is currently the only known pathogenic thermally dimorphic fungus in the genus *Penicillium*. Specifically, it forms white or brownish yeast-like colonies at 36 °C, while it grows in a mold form at 28 °C. Characteristically, the colonies of *T. marneffei* exhibit a “broom-like” structure, and produce a soluble wine-red pigment that diffuses into the agar at 28 °C [2]. *T. marneffei* infection can be classified into localized and disseminated types, which are associated with the functional status of the host immune system. Disseminated *T. marneffei* infection usually involves the liver, spleen, lymph nodes, intestines, and soft tissues, resulting in a high mortality rate [3].

*T*.*marneffei* is generally thought to be the most common opportunistic fungal infection among acquired immune deficiency syndrome (AIDS) patients in South China and Southeast Asia [4]. In recent years, with the development of organ transplantation, the widespread use of immunosuppressive agents, and the continuous exploration of related autoantibodies or gene mutations, the immunodeficient population has increased significantly, leading to a growing incidence of talaromycosis caused by *T. marneffei* infection. However, missed diagnosis and misdiagnosis are not uncommon because talaromycosis is rare with occult onset, diverse clinical manifestations and diverse radiographic manifestations [5]. Moreover, talaromycosis is often associated with many complications, resulting in complex treatment and serious side effects. Hence, the prognosis of *T. marneffei* is usually poor, with rapid progression and a high mortality rate. Here, we report a case of disseminated *T. marneffei* infection in an HIV-negative renal transplant recipient from non-endemic area with an initial presentation of skin rash and subcutaneous nodule for the first time. It’s also the second case of *T. marneffei* infection in a kidney transplant recipient who developed rare gastrointestinal bleeding. Additionally, we summarized the characteristics of *T. marneffei* in diagnosis and treatment, and especially presented the dangerous gastrointestinal bleeding in *T. marneffei*-infected kidney transplant patients in combination with the relevant literature.

## Case presentation

A 54-year-old male was admitted to the hospital on August 6, 2022, due to swelling and pain in his right hand after a crushing injury. The patient reported that over a year ago, he gradually developed scattered rashes and subcutaneous nodules throughout his body, mainly concentrated on the head, face, abdomen, and right upper limb. The patient had neither fever, cough, or sputum production nor abdominal pain, diarrhea, headache, chest pain, or hemoptysis. The patient had undergone renal transplantation 11 years prior due to uremia induced by idiopathic membranous nephropathy and was chronically treated with immunosuppressive agents and steroids after surgery. The current dosage was tacrolimus 1 mg qm, 0.5 mg qn; prednisone 2 tablets qd; and mycophenolate mofetil (MMF) 1 capsule tid. The patient had a history of hypertension and diabetes but no history of AIDS or tuberculosis. The patient had a 10-year history of hepatitis B and had been undergoing long-term antiviral treatment with adefovir dipivoxil at a dosage of 10 mg per day.

**Fig. 1:**
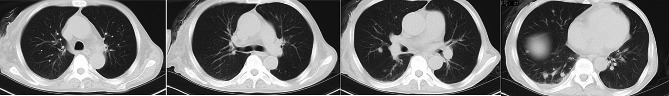
A chest computed tomography scan showed multiple pulmonary nodules in the bilateral lungs, inflammation in the bilateral lower lobes and thickened pleura in the right side. Mediastinal lymph nodes were not obviously enlarged

The breath sounds were coarse in both lungs, and a few wet rales could be heard in the right lower lung. There were no murmurs or additional heart sounds in the auscultation areas of each valve. Subcutaneous nodules were scattered throughout the body and partially ulcerated. There was no palpable enlargement of the superficial lymph nodes, bilateral tonsils, subcostal liver/spleen, or percussion pain in the bilateral renal regions. A lung computed tomography (CT) scan showed multiple nodules in the bilateral lungs, inflammation in the bilateral lower lobes, and thickened pleura in the right side. Mediastinal lymph nodes were not obviously enlarged (Fig. 1).

Blood routine test results suggested white blood cell (WBC), 11.43 × 10^9^/L; hemoglobin (Hb), 97 g/L; and C-reactive protein (CRP), 17.01 mg/L. Abnormal biochemistry results were as follows: alanine aminotransferase (ALT), 5 U/L; aspartate aminotransferase (AST), 7 U/L; prealbumin (PA), 115 mg/L; urea, 23.6 mmol/L; blood creatinine (Cr), 251 µmol/L; glucose, 12.8 mmol/L. Abnormal coagulation routine test results were as follows: von Willebrand Factor (vWF), 334%; Antithrombin III (ATIII), 76%.

**Fig. 2:**
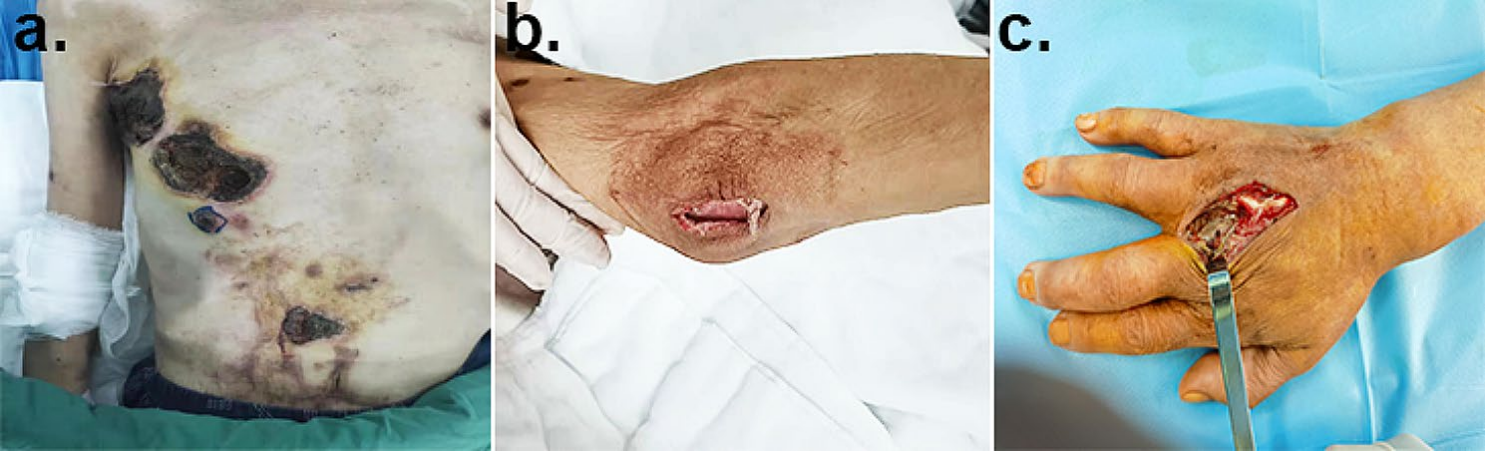
Preoperative photograph showing multiple ulcerations on anterior chest and abdomen before abscess incision drainage (**a**). Surgical fasciotomy on the right elbow (**b**) and the right hand (**c**) joint for decompression

**Fig. 3:**
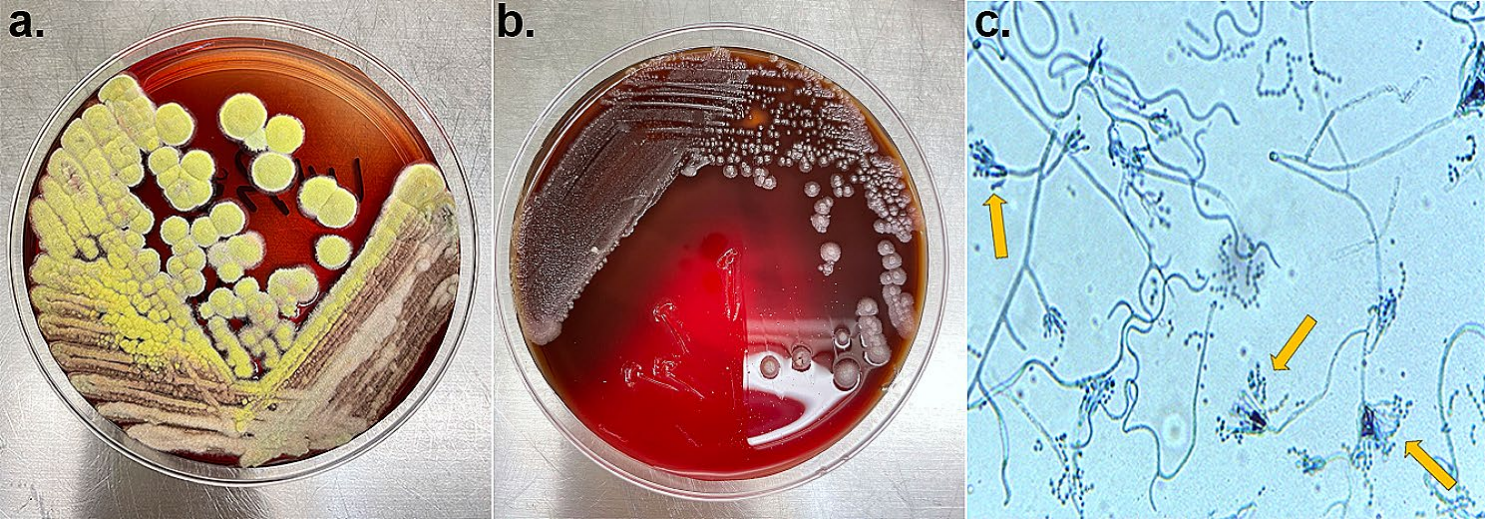
Bacterial culture of the purulent juice from three affected areas (right hand, right elbow and chest) revealed the presence of *T. marneffei*. (**a**). yellow villous colonies with characteristic red pigment production were observed after incubation on sabouraud dextrose agar at 28 ºC. (**b**). culture of *T. marneffei*. at 35 °C showed yeast phase on blood agar plate. (**c**). Light microscopy photo of lactophenol cotton blue staining from fungal purulent juice culture demonstrating brush-shaped hyphae (yellow arrow) in a smear preparation of colony culture at 28 ºC

**Fig. 4:**
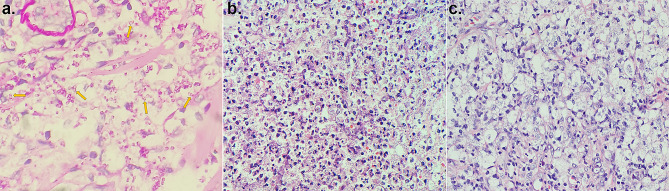
Periodic acid-schiff (PAS) staining of the chest wound tissue showing sausage-like spores with a central septum (yellow arrow) (**a**). Hematoxylin-eosin (HE) staining results showing chronic suppurative inflammation (**b**), foam cell aggregation and focal necrosis (**c**)

According to the imaging report (necrotizing fasciitis of the right hand and right elbow, purulent arthritis of the right hand, soft tissue infection of the right chest wall), the patient received abscess drainage, fasciotomy and decompression surgery on August 8th (Fig. 2). At the same time, the ruptured tissue was sent for bacterial culture and identification. The incubation was at 35℃, using blood agar and MacConkey agar. On August 9th, a morphology of mycelia was observed, and lactophenol cotton blue staining suggested “*Penicillium*”, which we considered a contaminant. Due to the patient’s skin rupture and exposure to air, other bacterial or fungal infections could not be ruled out. Subsequently, the cultivation of fungi was on sabouraud dextrose agar at 28℃ and 35℃ simultaneously. Later, the colonies incubated at 28℃ turned red. Although *T. marneffei* infection in a non-HIV renal transplantation recipient from a nonendemic area is rarely observed, this still raised our concern. Thus, we sent samples from multiple affected areas of the patient (hands, elbows, chest) for microbiological testing on August 15th. The pathogen growed in the filamentous form at 28℃ and in the yeast form at 35℃, which confirmed *T. marneffei* infection (Fig. 3), which was consistent with the mass spectrometry results. The chest histopathological results showed chronic suppurative inflammation with a large number of yeast-like cells with central septa, foam cell aggregation, and focal necrosis (Fig. 4), which further confirmed the result of the microbiological identification.

The laboratory features were getting worse, with a CRP level of 149.18 mg/L; a procalcitonin (PCT) level of 6.65 ng/ml; a hemoglobin (Hb) level of 65 g/L; a platelet count of 101 × 10^9^/L. The coagulation routine test results revealed prothrombin time (PT), 18.1s; activated partial thromboplastin time (APTT), 45.2s; prothrombin activity (PTA), 47%; and D-Dimer, 1.11 µg/mL. Considering the elevated neutrophils and C-reactive protein (CRP) level, piperacillin tazobactam (4.5 g/8 h) was given for antibacterial treatment. Due to the severity of the infection, oral prednisone was discontinued, tacrolimus and MMF remained at original dosage. Amphotericin B deoxycholate (initial dose: 0.5 mg/kg/d, therapeutic dose: 1.0 mg/kg/d) was given for antifungal treatment. On August 24th, the patient had melena (approximately 800 mL/d) and was diagnosed with gastrointestinal bleeding. This might have been caused by *T. marneffei* involvement in the gastrointestinal mucosa. At the same time, the side effects of amphotericin B in the gastrointestinal tract were also related [6, 7]. On the night of August 25th, the patient experienced abdominal pain. Emergency abdominal CT revealed free gas in the abdominal cavity and gas density shadows around the liver and abdominal cavity, indicating gastrointestinal perforation and acute peritonitis. The patient underwent surgical repair of the intestine. The antibiotics were changed to ertapenem (1 g, qd), and Adefovir was discontinued. Over the next two days, the platelet count gradually decreased, and the levels of CRP and procalcitonin (PCT) were abnormally elevated. However, the serum creatinine level remained between 206 and 260 µmol/L, and the liver function indicators aspertate aminotransferase (AST) and alanine aminotransferase (ALT) did not significantly increase. The dose of MMF was adjusted to 1 capsule bid. Furthermore, maltose-fermenting *Pseudomonas aeruginosa* was cultured from sputum, *Burkholderia cepacia* was cultured from blood, and *Enterobacter cloacae* and *Pseudomonas aeruginosa* were cultured from ascites. Subsequent blood metagenomic next-generation sequencing (mNGS) reported *T. marneffei* (341), cytomegalovirus (153), parvovirus (43), herpes simplex virus 1 (HSV1) (15), and epstein-barr virus (EB) (16). Clinically, ertapenem was replaced, and meropenem and ganciclovir were used for antibacterial and antiviral treatment, respectively. The dose of MMF was adjusted to 1 capsule qd for nasal feeding. Unfortunately, the patient soon developed acute respiratory failure, multiple organ dysfunction syndrome (circulatory, renal, hematological, coagulation), shock and hepatic encephalopathy. Finally, the patient’s family members abandoned treatment and requested discharge. One month later, during follow-up, the patient died after failed cardiopulmonary resuscitation.

## Discussion and literature review

As one of the most common opportunistic infection in patients with AIDS, *T. marneffei* infection has been increasingly reported as a coinfection in non-HIV populations, such as those with malignancy, organ transplantation, autoimmune diseases, etc. Due to the different mechanisms of immunodeficiency in *T. marneffei* patients, the clinical manifestations are often varied and complex. The most common symptoms include fever (82%), skin lesions (40.7%), hepatosplenomegaly (32%), and lymphadenopathy (33.3%). In addition, approximately one-third of patients experience gastrointestinal symptoms such as diarrhea [8]. Due to the lack of specificity in clinical indicators, the misdiagnosis rate is high. Furthermore, talaromycosis is a dangerous and rapidly progressive disease with a high mortality rate because *T. marneffei* can invade almost all systems and organs, including the lungs, liver, bones, and blood, and the side effects of treatment drugs are severe.

### Difficulty in and development of strategies for diagnosing *T. marneffei* infection

Currently, positive pathogen culture is the gold standard for diagnosing *T. marneffei*. However, early pathogen diagnosis has a low positive rate, often leading to delayed treatment, misdiagnosis, or even incorrect treatment. The main reasons for this are as follows: (a) given that patients often have negative blood cultures in the early stage, cultivation and identification from different sites, such as the bone marrow and lymph nodes, is neglected, especially in patients without rash, who always have a higher risk of delay, and (b) a blue fungus that produces a wine-red pigment is not necessarily *T. marneffei*, and its identification requires observing the colony morphology and the conversion of mold to yeast, which takes a long time. In addition, there are other auxiliary diagnostic methods, such as detecting serum galactomannan (GM) antigen, but it has cross-reactivity with *Aspergillus* infection and low specificity. Histologically, H&E staining can be used, but due to the similarity between this fungus and the capsule variant form of *Histoplasma capsulatum*, it is often mistaken for having a capsule, leading to misjudgment. Although it cannot be used as a definitive pathogen diagnosis, molecular biology detection has become an important method for fungal infection pathogen detection in recent years. The use of PCR amplification technology to detect fungal DNA is helpful for early identification, such as detecting the *T. marneffei* cell wall-specific polysaccharide antigen galactomannan protein Mp1p, which has good application prospects [9, 10]. In recent years, the rapidly developing mNGS technology has also shown significant advantages in identifying *T. marneffei*. It does not require cultivation, saving a significant amount of time, and has high diagnostic sensitivity and specificity [11, 12]. mNGS can also be used to identify rare fungi and other types of pathogens, providing rapid diagnostic value. Additionally, some scholars have applied ultrahigh-performance liquid chromatography‒mass spectrometry (LC‒MS) to detect serum biomarkers in *T. marneffei* patients. The research results show that HIV-negative *T. marneffei* patients have abnormal metabolism of sphingolipids, and a serum level of Sa (d16:0) at 302.71 nM has a sensitivity of 87.5% and specificity of 100% for diagnosis [13].

### Pathogenesis and symptoms of talaromycosis

Immune deficiency causes the inability of macrophages to effectively clear ingested *T. marneffei*, leading to the abnormal proliferation of such macrophages and systemic disseminated infection through lymphatic and blood circulation [14–16].

In addition to HIV or long-term use of immunosuppressive drug caused immune deficiency, autoantibodies or gene mutations relevant immune deficiency has also been gradually discovered and valued in *T. marneffei* infection. Guo et al. found a high positive rate, up to 94.8%, of anti-INF-γ autoantibodies in a population of 58 HIV-negative *T. marneffei*-infected individuals, and plasma from patients positive for anti-INF-γ autoantibodies impaired the clearance of *T. marneffei* by THP-1 cells [17]. High titers of anti-INF-γ autoantibodies can also inhibit the phosphorylation of STAT1 and Th1 cell differentiation in CD4 + T cells [18]. Patients with primary immunodeficiency diseases (PID) caused by STAT1 and/or STAT3 gene mutations often have defects in T/B/NK cells’ function and INF-γ production, and they are also a high-risk group for *T. marneffei* infection [19–22]. In addition, there are reports of *T. marneffei* infection in patients with CARD9 gene mutations [23, 24], CD40 ligand deficiency [25–27], RelB deficiency [28], IL-2 receptor common γ chain deficiency, and adenosine deaminase deficiency [29]. The above reports mainly involve T-lymphocyte-activation-related signaling pathways and the NF-κB signaling pathway.

*T. marneffei* mainly invades the lungs and then spreads to the liver, spleen, etc. It is also possible to invade through the digestive tract or skin wounds by contact with water or soil contaminated with spores. Therefore, the primary symptoms of talaromycosis are mostly fever and cough, occasionally with gastrointestinal symptoms such as diarrhea as the initial symptoms [30]. In this case, the patient gradually developed scattered skin rashes and subcutaneous nodules starting in 2021. However, due to the absence of common symptoms of talaromycosis (cough, fever, diarrhea, hepatosplenomegaly, etc.) and the conventional understanding that *T. marneffei* infection is rarely observed in cold, northeastern China as well as blood and skin lesion cultures that did not show positive bacteria, *T. marneffei* failed to be identified and treated in the early stages of infection. The lack of clinical suspicion leading to delay or misdiagnosis is an important reason why the mortality rate of HIV-negative *T. marneffei*-infected patients is higher than that of HIV-positive patients [31]. Multiple skin lesions, subcutaneous nodules, or abscesses are usually an early manifestation of these disseminated cases. If *T. marneffei* was considered early in these cases, biopsy could be performed at multiple skin lesion sites or follow-ups could be conducted, which might lead to an early diagnosis and have important significance for timely clinical treatment and improved prognosis.

### Clinical immunosuppressive and therapeutic regimen for non-HIV transplant patients with *T. marneffei* infection

Currently, cases of *T. marneffei* infection in non-HIV organ transplantation recipients are still rare. Considering the particular immunosuppressive therapy, the treatment of *T. marneffei* in these patients is a real challenge. So, we summarized the relevant immunosuppressive and therapeutic regimen here. We searched literatures in the PubMed databases using the terms “Talaromyces marneffei” AND “Transplant”, a total of 12 HIV-negative cases that we could access full text were retrieved till date (Table 1) [32–42]. The majority was kidney transplant recipients (*n* = 10) followed by lung transplant (*n* = 2). Six of the 12 patients had coughing and fever as the first symptom, 4 had abdomen and back pain or headache, 2 had diarrhea, 2 had elevated serum creatinine, and only 1 had sore throat and odynophagia. The routine medications after transplantation include tacrolimus (Tac), MMF, prednisolone or Methylprednisolone (Methylpred). After diagnose of *T. marneffei* infection, MMF was discontinued in 5 of the 12 patients, and reduced in 3 cases. Methylpred was seldom adjusted in almost all cases, excepting cessation in 2 patients. The most frequently reduced or even discontinued immunosuppressant is Tac, because when using voriconazole and itraconazole to defend against *T. marneffei* infection, the metabolism of Tac by CYP enzyme can be significantly inhibited, leading to the increased blood concentration of Tac. Hence, close monitoring of tacrolimus concentration and timely dose adjustments are necessary. Most of the cases stated Tac was adjusted according to its blood concentration. Currently, the optimal therapeutic window for tacrolimus in kidney transplant patients is 3–6 µg/L starting from 12 months after surgery. However, there is no consensus standard or recommendation for adjusting tacrolimus concentrations in transplant patients with invasive fungal infection, especially those with severe infection. Temporarily discontinuing Tac was reported in cases of severe infection [30]. This would require systematic evaluation of the patient.

**Table 1:** Clinical immunosuppressive and therapeutic regimen for non-HIV transplant patients with *T. marneffei* infection

Age(yr)/gender	Area	Transplant-ed organs	Initial presentation	Immunosuppressive agent adjustment	Treatment	Outcome	Ref.
33/M	Zhejiang, China	kidney	left lower abdomen and back pain	After admission: Tac (1 mg bid to 0.5 mg bid); MMF was stopped	Itraconazole (200 mg bid)	Cured	Luo S, et al. [32], 2023.
31/M	Hainan, China	kidney	low back pain with intermittent diarrhea	After admission: Methylpred (80 mg qd); other anti-rejection drugs was stopped	Voriconazole (200 mg bid); amphotericin B cholesteryl sulfate complex (4 mg/kg)	Cured	Xu L, et al. [30], 2023.
54/M	Shen zhen, China	kidney	sore throat and odynophagia	Tac dose was adjusted according to its blood concentration	Voriconazole (200 mg bid)	Cured	Pan M, et al. [33], 2023.
47/F	Chang sha, China	kidney	elevated serum creatinine	After diagnose of talaromycosis: Tac (1 mg bid to 0.5 mg bid); Tac dose was adjusted according to its blood concentration	Voriconazole (200 mg bid); then changed to itraconazole (200 mg/day)	N/A	Li Y, et al. [34], 2022.
51/M	Jiangxi, China	kidney	coughing, fever	After admission: MMF (750 mg qd, 1000 mg qn to 250 mg bid); Tac dose was adjusted according to its blood concentration	Voriconazole (200 mg bid)	Cured	Cai DH, et al. [35], 2022.
61/M	Guangxi, China	kidney	coughing, fever and expectoration	After admission: Glucocorticoids were stopped; Tac was adjusted according to its blood concentration	Voriconazole (200 mg bid)	N/A	Xing S, et al. [36], 2022.
55/M	Guangxi, China	kidney	fever and shortness of breath	After admission: Tac, MMF and prednisolone were discontinued; Methylpred (80 mg qd)	Voriconazole	Died	
51/M	Sichuan, China	kidney	coughing, hemoptysis	After admission: MMF (0.5 g bid); Tac (1.0 mg bid)	Oral posaconazole (400 mg bid)	Cured	Lang Q, et al. [37], 2020.
53/F	England	kidney	coughing, green sputum	MMF was discontinued; Tac dose was reduced	Liposomal amphotericin B daily for 2 weeks; oral itraconazole twice daily for 6 months	Cured	Vergidis P. et al. [38], 2021. Ma W, et al. [39], 2018.
51/M	Guang dong, China	kidney	serum creatinine elevation	After admission: Tac and Methylpred were stopped; MMF was withheld; CsA (concentration ranging from 140.6 to 503.8 ng/mL)	Liposomal amphotericin B (0.4 mg/kg/day); itraconazole (200 mg bid)	Cured	Peng J, et al. [40], 2017.
61/M	Belgium	Lung	fatigue, fever, oral ulcers, anorexia, diarrhoea and abdominal pain	N/A	Liposomal amphotericin-B (3 mg/kg/day); oral voriconazole (4 mg/kg bid, maintenance therapy)	Cured	Hermans F, et al. [41], 2017
41/F	Australia	Lung	fever, wheezing, reduced exercise tolerance, and headache	After admission: MMF was stopped; Tac (4 mg bid to 1.5 mg qd), Tac was adjusted according to its blood concentration	Prophylactic intravenous voriconazole (loading dose 370 mg qid, maintenance dose 250 mg bid); oral voriconazole (200 mg bid, after identifying talaromycosis)	Cured	Stathakis A, et al. [42], 2015.

Abbreviations: Tac: tacrolimus; MMF: mycophenolate mofetil; Methylpred: Methylprednisolone;

N/A: not available

In terms of *T. marneffei* infection treatment, the current recommended therapeutic regimen is as follows: intravenous administration of liposomal amphotericin B (3–5 mg/kg/day), amphotericin B lipid complex (5 mg/kg/day), or amphotericin B deoxycholate (0.7 mg/kg/day) for 2 weeks, followed by consolidation therapy with oral itraconazole 200 mg bid for 10 weeks. If itraconazole is not tolerated, voriconazole can be taken orally, with a starting dose of 400 mg bid on the first day, followed by 200 mg bid for a total of 12 weeks. Subsequent maintenance therapy uses itraconazole 200 mg qd, orally, or voriconazole 200 mg bid, orally.

Although amphotericin B has better treatment effects against talaromycosis, its significant nephrotoxicity is a clinical concern, especially for kidney transplant patients. The nephrotoxicity of amphotericin B is related to its ability to damage the permeability of cell membranes, leading to excessive entry of calcium ions into cells, resulting in cell death [43]. Additionally, coadministration of amphotericin B and tacrolimus will also increase renal toxicity. Therefore, in clinical practice, voriconazole or itraconazole is usually chosen as the preferred treatment.

### Dangerous gastrointestinal bleeding in *T. marneffei*-infected kidney transplant patients

Another clinical concern is gastrointestinal bleeding caused by involvement of the *T. marneffei* gastrointestinal mucosa or drug side effects. Although gastrointestinal ulcers and erosion are common in *T. marneffei*-infected patients (∼ 31%), gastrointestinal bleeding is very rare. It is worth noting that even among the most common HIV-positive patients with *T. marneffei* infection, though about a third of these patients have symptoms such as ulcers, only 2 cases of gastrointestinal bleeding could be officially searched using the Pubmed electronic database [44, 45]. However, among 11 *T. marneffei*-infected kidney transplant patients, including the patient described in the current case, 3 patients had gastrointestinal bleeding [30, 32], accounting for 27.3%, which is much higher than the proportion of gastrointestinal bleeding in HIV patients. Moreover, three of the above four patients experienced hemorrhagic shock, which is is usually fatal. Therefore, more attention should be given to the occurrence of gastrointestinal bleeding in kidney transplant patients after *T. marneffei* infection.

Gastrointestinal bleeding in these patients occurs for the following three reasons: (a) the side effects or adverse reactions of glucocorticoid drugs, especially regarding upper gastrointestinal bleeding; (b) stress ulcers caused by severe trauma, critical illness, or severe psychological disorders; and (c) gastrointestinal side effects of calcineurin inhibitor drugs (e.g., tacrolimus, cyclosporine), although these drugs rarely cause gastrointestinal bleeding. Therefore, if *T. marneffei* infection affects the gastrointestinal tract, the risk of gastrointestinal bleeding in kidney transplant patients may be significantly increased. Moreover, once it occurs, it can easily become recurrent gastrointestinal bleeding [30]. Conventional surgical treatment is not ideal, and bleeding may still occur in other parts of the intestines after surgery. Additionally, endoscopic hemostasis treatment carries the risk of causing or exacerbating bleeding, which often proves fatal if it leads to hemorrhagic shock [30, 44]. Therefore, early diagnosis and timely treatment are necessary for kidney transplant patients suspected of having *T. marneffei* infection.

Among the two *T. marneffei*-infected kidney transplant cases, including our case, patients started using fluconazole or amphotericin B approximately 12 days after admission, and they all experienced gastrointestinal bleeding [30]. On the other hand, other renal transplant patients who had timely confirmed *T. marneffei* infection through mNGS after admission and started taking medications within 5 days did not show significant gastrointestinal symptoms, and the treatment process went relatively smoothly [35–37]. Hence, early mNGS or other molecular biology tests (such as Mp1p assessment) should be conducted on transplant patients with unexplained fever, cough, rash, abdominal pain, and diarrhea, and prophylactic medication should be implemented to prevent disease deterioration and improve prognosis.

## Conclusions

The incidence of invasive fungal infections such as *T. marneffei* infection has been increasing year by year due to long-term use of immunosuppressive drugs after kidney transplantation as well as the spread of pathogenic microorganisms caused by population movement and the genovariation of the pathogens themselves [46–48]. Thus, we absolutely cannot ignore the possibility of *T. marneffei* infection in non-HIV patients in nonendemic areas. What’s more, the risk of fatal gastrointestinal bleeding can be significantly increased in kidney transplant patients with *T. marneffei* infection because of the long-term side effects of post-transplant medications. Hence, strengthening clinical awareness and using mNGS, mass spectrometry, and other technologies to improve the detection rate and early diagnosis of *T. marneffei* are crucial for clinical treatment. Balancing antifungal therapy, immunosuppression, and preventive measures against bleeding to achieve the safest and most effective treatment for patients requires tailored and rational personalized treatment plans based on individual patient conditions.

## Data Availability

No datasets were generated or analysed during the current study.

## Abbreviations

AIDS: Acquired immune deficiency syndrome
ALT: Alanine aminotransferase
AST: Aspertate aminotransferase
CRP: C-reactive protein
CT: Computed tomography
EB virus: Epstein-barr virus
GM: Galactomannan
HIV: Human immunodeficiency virus
HSV1: Herpes simplex virus 1
LC-MS: Liquid chromatography-mass-spectrometry
mNGS: Metagenomic next-generation sequencing
PCT: Procalcitonin
PID: Primary immunodeficiency disease
TCR: T-cell receptor
T. marneffei: Talaromyces marneffei
